# Supplementary figures and images for: Temporal regulation of MDA5 inactivation by Caspase-3 dependent cleavage of 14-3-3η
Source: PLoS Pathog. 2024 Jun 6;20(6):e1012287. doi: 10.1371/journal.ppat.1012287 (PMC11185488; doi:10.1371/journal.ppat.1012287)

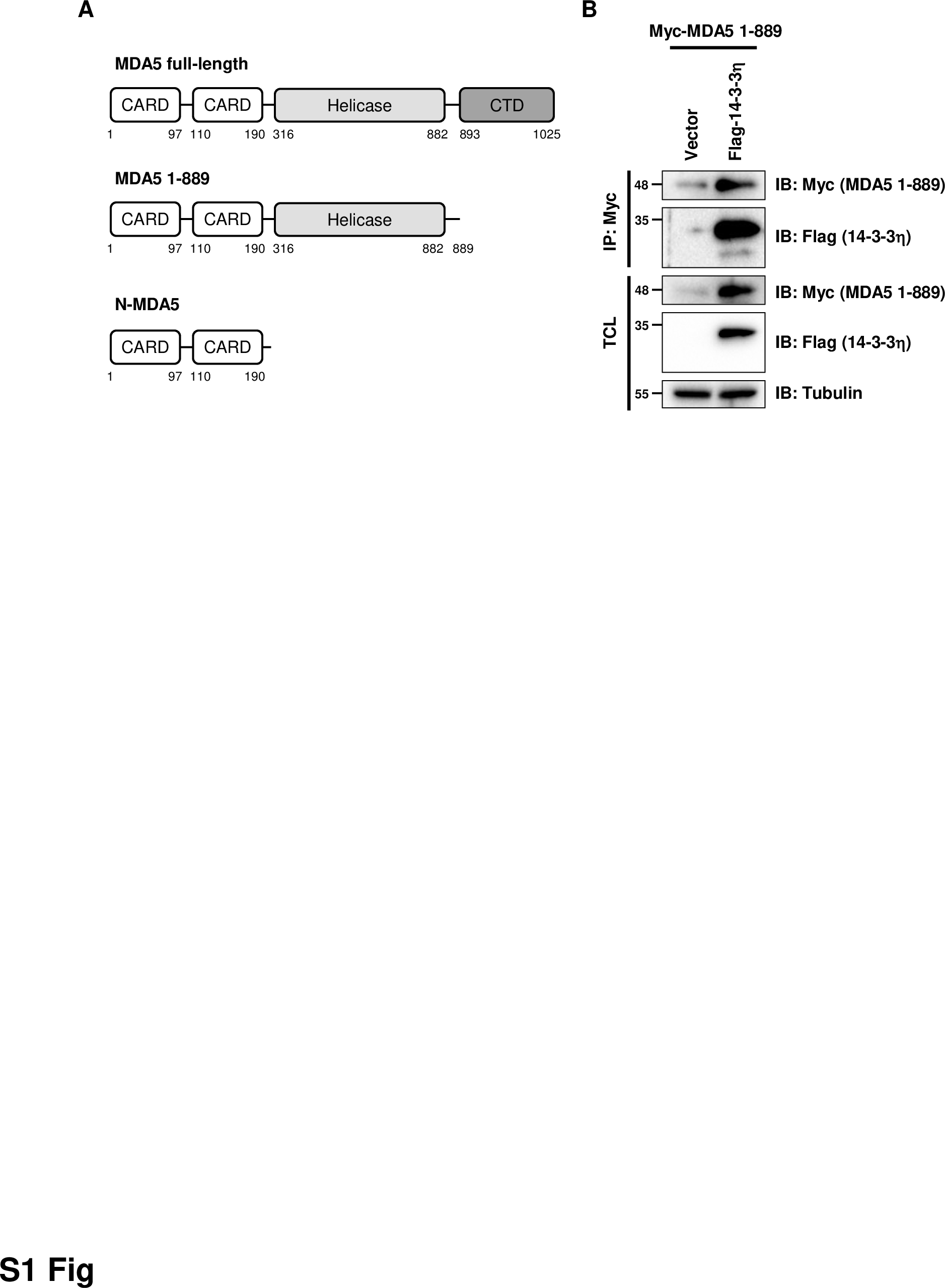

Supplement: S1 Fig — (A) The schematic diagram of different MDA5 constructs. (B) Myc-MDA5 1–889 was co-transfected with empty vector or Flag-14-3-3η into Huh7 cells, followed by the anti-Myc immunoprecipitation (IP) to determine the Flag-sub-14-3-3η accumulation. (TIF) [file ppat.1012287.s001.tif]

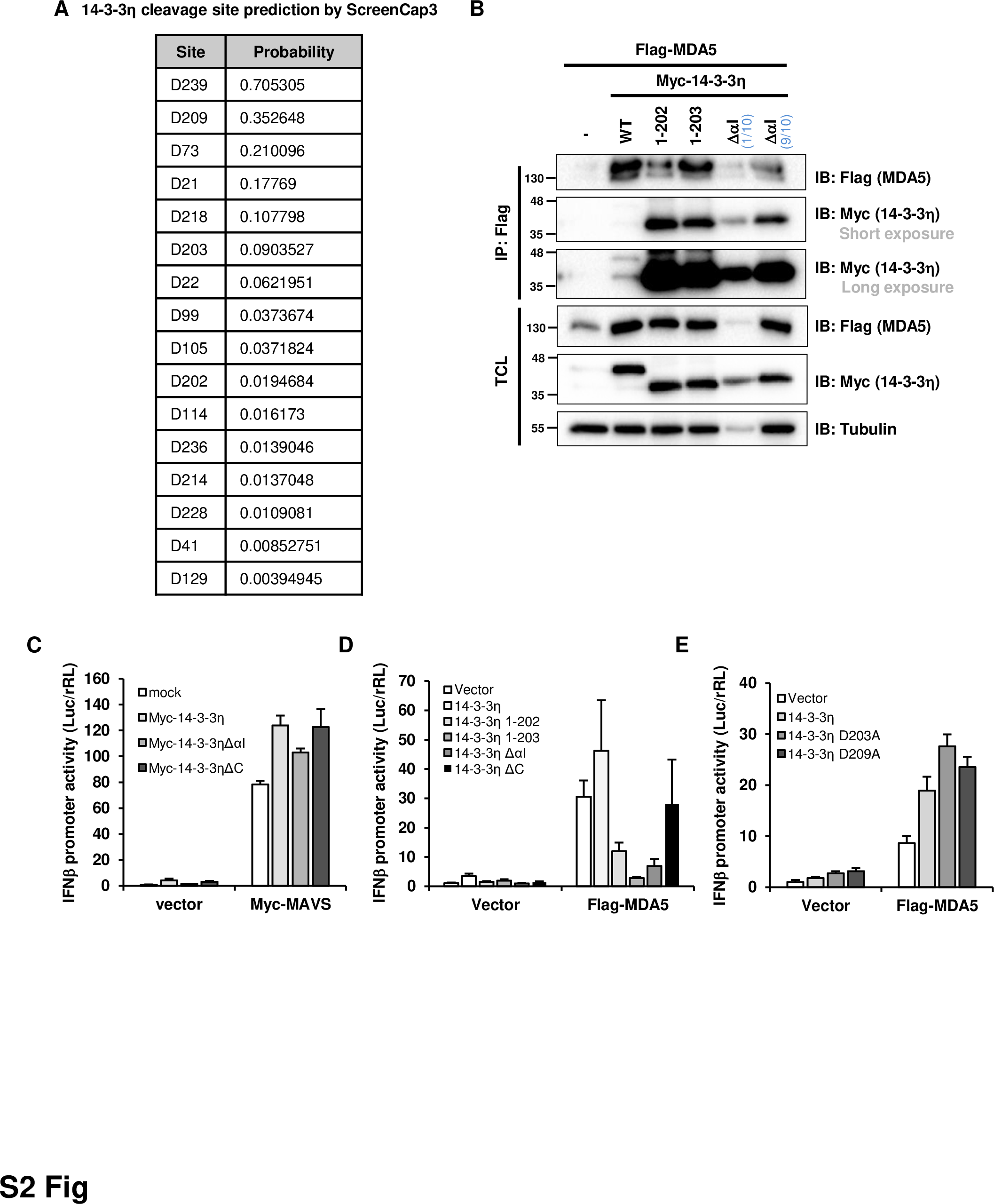

Supplement: S2 Fig — (A) Prediction of Caspase-3 cleavage sites of 14-3-3η via ScreenCap3 database. The probability score ranged from 0 to 1. The higher score meant the more probability of the Caspase-3 cleavage. (B) Huh7 cells were co-transfected with Flag-MDA5 and Myc-14-3-3η wildtype or truncated mutants for 48 hours, followed by anti-Flag immunoprecipitation (IP) to determine the interactions of Flag-MDA5 and Myc-14-3-3η wildtype or truncated mutants. (C) Indicated Myc-14-3-3η constructs were co-transfected with empty vector or Myc-MAVS into Huh7 cells and then cell lysates were detected the IFNβ promoter activities by dual luciferase reporter assay. (D) to (E) Myc-14-3-3η wildtype, (D) truncated mutants, or (E) indicated D to A mutants were co-transfected with empty vector or Flag-MDA5 into Huh7 cells. Dual luciferase reporter assay was performed to monitor the IFNβ promoter activities of cell lysates. (TIF) [file ppat.1012287.s002.tif]

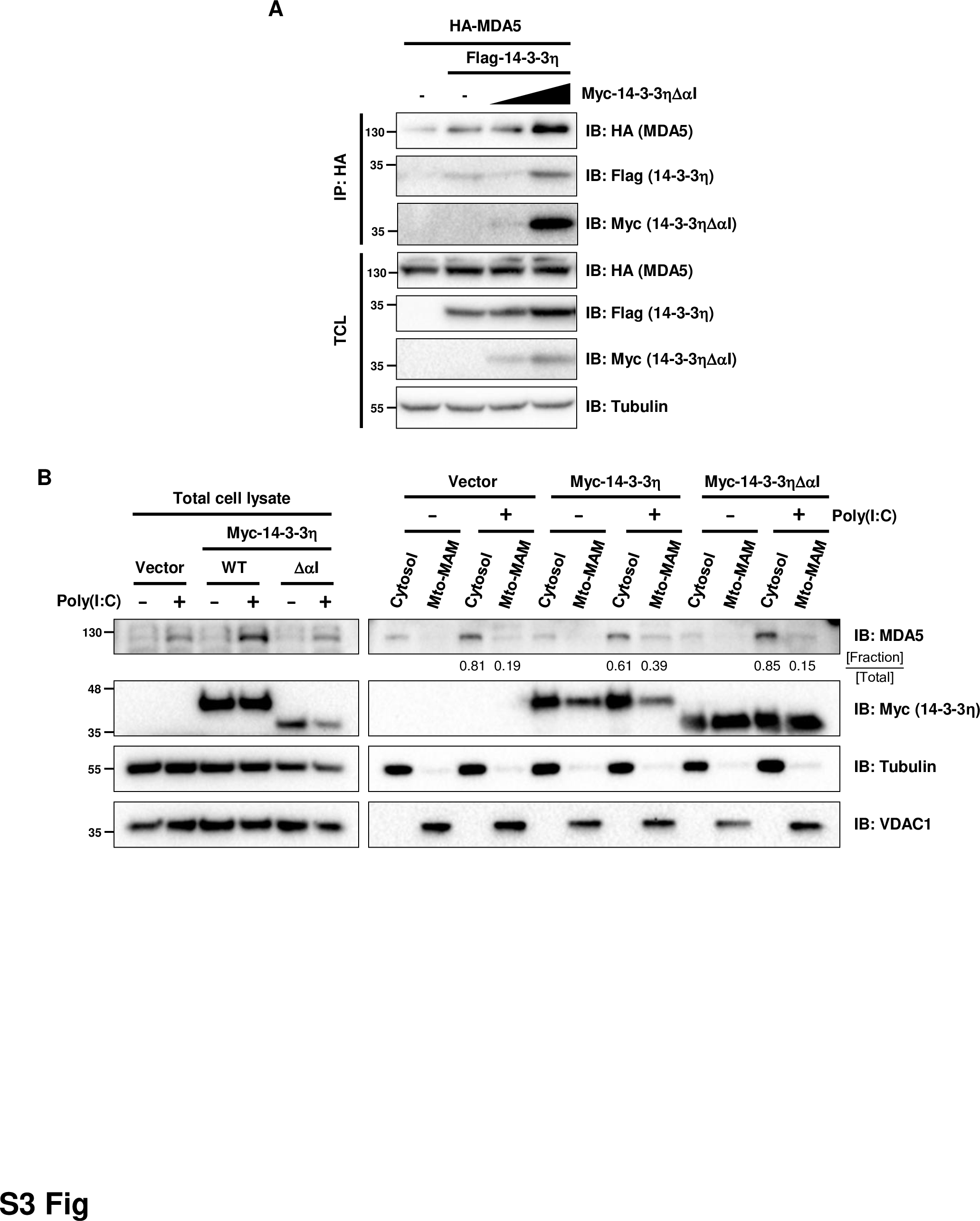

Supplement: S3 Fig — (A) HA-MDA5, Flag-14-3-3η and Myc-14-3-3ηΔαI were co-transfected into Huh7 cells. Anti-HA immunoprecipitation (IP) was performed to determine the interaction of the ectopic expressing proteins. (B) 14-3-3η KD Huh7 cells were transfected with empty vector, Myc-14-3-3η or Myc-14-3-3ηΔαI for 24 hours, followed by mock-transfection or 1 μg/mL HMW poly(I:C) transfection for 18 hours. Cell lysates were separated into cytosol and mito-MAM fractions. Immunoblotting was utilized for detecting the redistribution of endogenous MDA5. (TIF) [file ppat.1012287.s003.tif]

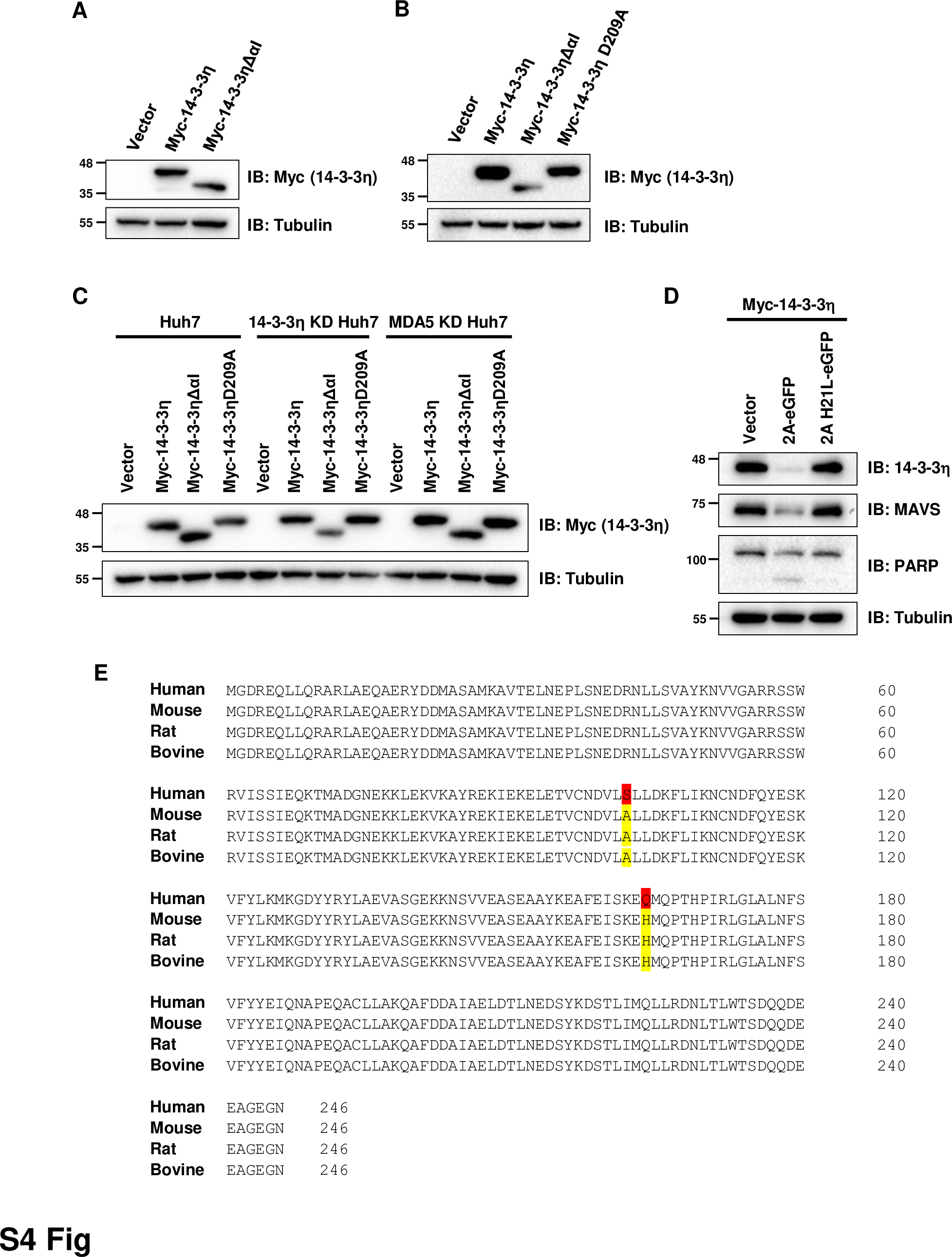

Supplement: S4 Fig — (A) Continued to Fig 4A, immunoblotting was used to confirm the ectopic expression. (B) Continued to Fig 4B, immunoblotting was used to confirm the ectopic expression. (C) Continued to Fig 4C and 4D, immunoblotting was utilized to confirm the ectopic expression in Huh7 cells, 14-3-3η KD Huh7 cells and MDA5 KD Huh7 cells. (D) Constant amount of Myc-14-3-3η and empty vector, EV71 2A-eGFP or EV71 2A H21L-eGFP were co-transfected into Huh7 cells for 72 hours. The protein levels were analyzed by immunoblotting. (E) Whole amino acid residues of 14-3-3η from different species were aligned. The sequences of 14-3-3η across different species were highly conserved. (TIF) [file ppat.1012287.s004.tif]
