# Supplementary material for: Temporal regulation of MDA5 inactivation by Caspase-3 dependent cleavage of 14-3-3η
Source: PLoS Pathog. 2024 Jun 6;20(6):e1012287. doi: 10.1371/journal.ppat.1012287 (PMC11185488; doi:10.1371/journal.ppat.1012287)
Supplement: Attachment — Submitted filename: paper response HL1Rui1HML2.docx [file ppat.1012287.s006.docx]

**Response to editors and reviewers:**

**Comment:**

Thank you for submitting your manuscript to PLoS Pathogens. After a thorough review by two referees, we have reached the decision to request a major revision of your manuscript.

Cleavage of 14-3-3η by Caspase-3 during MDA5 activation produces sub-14-3-3η, impairing antiviral innate immunity, suggesting an interesting hypothesis by which RNA viruses may antagonize host defenses by activating cell death. Both innate immune responses and death are important issues for a host cell response to invading pathogens and contribute to the infection's final outcomes. From a pathogen's point of view, innate immunity should be turned off first so that makes the infection and the cause of death easier. Does the virus really benefit from sub-14-3-3 to its infection? Why and how a pathogen wants to use a relatively late event of the infection (caspase activity) to deal with a relatively urgent issue (antagonizing innate immunity) is interesting and of which biological significance needs more discussion. Meanwhile, showing 14-3-3 as a direct substrate of the caspase is suggested to further strengthen the statement.

Upon reviewing the attached reports, you will note that the reviewers have raised concerns about the study and manuscript preparation that hampered the acceptance of this manuscript at the current stage. Despite we hope to see experiments showing that 14-3-3 cleavage does have an impact on virus infection, a biological explanation and discussion should be included, no matter whether the impact existed or not. Besides, scientific proofreading and grammar copyediting were also suggested. Consequently, we kindly request that you address these concerns through additional work.

**Response to Editor:**

Thanks to the editor’s summary. In our current study and other reports, it seems like the cleavage of 14-3-3η or 14-3-3 ε is a common theme in many different viral infections, and the cleavage can be achieved by either the host or the viral proteases (J Virol doi: 10.1128/jvi.00604-23). In the revised manuscript, we tested whether sub-14-3-3η has the functional role in viral replication (Fig. 4C). According to our results, the viral mRNA levels were higher in 14-3-3η KD cells and 14-3-3ηΔαI could not inhibit the viral replication. However, the viral replication was be suppressed by 14-3-3η WT and D209A transfected WT or 14-3-3η KD Huh7 cells. Additionally, transfection of 14-3-3η D209A in wildtype Huh7 cells showed a faster suppression of viral replication than those transfected with 14-3-3η WT, which was not observed in the MDA5 K/D Huh7 cells. These results suggest that the presence of sub-14-3-3η may promote viral infection.

In our proposed model, we hypothsize that when MDA5-mediated IFN-beta induction is activated, it will lead to the activation of Caspase-3. Caspase-3 cleaves 14-3-3η to form sub-14-3-3η which binds to MDA5 to turn off the signaling. In this way, over-activation of type I IFN induction could be prevented in cells. Therefore, though viruses have many strategies to antagonize host, viruses can utilize Caspase-3 activity to promote viral infection coincidentally. We have extended the discussion to the biological significance in caspase-3 dependent and independent expression of sub-14-3-3η.

As the Editor suggested, to confirm 14-3-3η is the direct substrate for the Caspase-3, we tried to perform the *in vitro* Caspase-3 cleavage assay with purified recombinant proteins. Within this limited revision time, we were not able to determine the best condition of *in vitro* Caspase-3 cleavage assay to shown a direct cleavage of 14-3-3η by Caspase-3. Our preliminary results suggested that recombinant 14-3-3η was not cleaved by recombinant Caspase-3 directly *in vitro* (data not included). It is likely that certain post-translational modifications of 14-3-3η and/or the presence of active MDA5 are/is required to facilitate 14-3-3η cleavage by Caspase-3.

**Response to Reviewer #1:**

**Reviewer #1:** Chan et al. describe the identification of a cleaved form of 14-3-3η and its plausible role in regulating MDA5 function. In a previous report, the authors have noted that a distinct band of 14-3-3η with faster electromobility, termed sub-14-3-3η, was observed in a co-immunoprecipitation assay of MDA5. In the current study, Chan et al. found that the expression of sub-14-3-3η was specific to the presence of caspase-3 and was upregulated by coronavirus and enterovirus infections. In addition, overexpression of sub-14-3-3η was shown to downregulate MDA5-mediated IFN induction and subsequent ISG expression, and the authors claimed that this was likely due to inhibition of MDA5 redistribution to mitochondria. Consistent with these findings, reduced IFN induction in response to virus infection was also observed in sub-14-3-3η-expressing cells.

The experiments presented were performed appropriately and the manuscript is easy to follow. The identification of a possible negative regulator of MDA5, which is generated from full-length 14-3-3η by Caspase-3, is novel. From an immunological point of view, the study would be interesting in terms of auto-feedback regulation of the innate immune response. However, with the data presented, the functional role of sub-14-3-3η in virus replication remains unclear.

**Response:**

Thanks Reviewer #1 for many suggestions for us to improve the manuscript. We have included new figures in Figure 2E, 4C and 4D, and detail responses were written under each comment.

**Major Issues:**

**Comment:**

1. The association of MDA5 with sub-14-3-3η and the redistribution of MDA5 should also be demonstrated by immunofluorescence analysis.

**Response:**

The proposed immunofluorescence analysis (IFA) experiment by reviewer was staightforward and logical. We would love to have that data as well, however, due to the limitation of antibody, we have not found a good anti-MDA5 antibody for endogenous MDA5 statining. With Flag-tagged MDA5, we could stain but also were unable to determine any specific pattern in the cytosol when comparing the intracellular distribution of Flag-MDA5 in the infected versus the uninfected cells. We think this is probably due to that only a small portion of MDA5 was activated and moved to mitochondia under poly (I:C) stimulation or virus infection. This phenotype was also observed by cytosol-mitochondria fractionation followed by immunoblotting (Fig. 2C and our previous reports in PLoS Path 2019 and Cell Host and Microbe 2012). Also, ectopically expressed MDA5 may often increase the basal levels of IFNβ mRNA without other stimulation. The other thing is that 14-3-3η intracellular distribution may also be affected by many other cargo proteins of 14-3-3η, and only a certain percentage is interacting with MDA5. Therefore, as much as we also would like to have a clear result of IFA, our IFA data so far (data not shown) could not determine the association of MDA5 and 14-3-3η or sub-14-3-3η.

**Comment:**

2. The weakest point of this paper is that it does not show a functional role for sub-14-3-3η in viral replication, thus one should question the submission to PLoS Pathogens. If sub-14-3-3η functions as a negative regulator of MDA5-mediated IFN induction even in virus-infected cells, expression of the caspase-3-resistant version of 14-3-3η (i.e. the D209A mutant) confers a higher antiviral property than the WT one. Also, virus replication should be enhanced by sub-14-3-3η expression. These data are crucial, I believe.

**Response:**

Thanks to the reviewers comment. We totally agree that for publications in PLoS Pathogens, the infection data is critical. We have tested several different infection conditions at different MOIs with different infection time. We finally have completed this experiment and included the results in **new Fig. 4C-D**. According to our results, the viral mRNA levels were higher in 14-3-3η KD cells and 14-3-3ηΔαI could enhance the viral replication. Furthermore, the viral replication was be suppressed by 14-3-3η WT and D209A transfected WT or 14-3-3η KD Huh7 cells. Additionally, transfection of 14-3-3η D209A showed a faster suppression of viral replication than those transfected with 14-3-3η WT, which is consistent to our IFNβ induction results. We also found that the phenotype was MDA5-dependent (Fig. 4D).

**Comment:**

3. Page 7, 1st line "a stronger binding affinity...": The data in Fig. 2B do not clearly demonstrate that the affinity of sub-14-3-3η for MDA5 is "stronger" than that of full-length 14-3-3η. A competition assay should be performed in which full-length and sub-14-3-3η are simultaneously transfected, followed by MDA5 immunoprecipitation.

**Response:**

The results of competition assay of full-length 14-3-3η and sub-14-3-3η have been showed at supporting information figure S3A. Our results showed that Flag-14-3-3η could be co-immunoprecipitated with HA-MDA5. When Myc-14-3-3ηΔαI was dosed into the anti-HA co-IP experiment, we can see that the interaction of Flag-14-3-3η and HA-MDA5 was reduced with low expression of Myc-14-3-3ηΔαI and was enhanced with high Myc-14-3-3ηΔαI abundance, suggesting that competition between full-length 14-3-3η and sub-14-3-3η to MDA5 interaction may not be the only mechanism for sub-14-3-3η to control MDA5 signaling.

**Minor Issues:**

**Comment:**

1. Molecular weight mass should be indicated on all immunoblotts.

**Response:**

Thank the reviewer for the suggestion. We added the molecular weight in our figures.

**Comment:**

2. Fig. 2E: Immunoblot of immunoprecipitated D209A by Myc antibody detection is unclear.

**Response:**

The new results with the clear bands have been updated in Fig 2E.

**Comment:**

3. Where is Fig.3D mentioned in the main body of text?

**Response:**

We are sorry about this mistake. Fig. 3D described the quatitative result of Fig. 3C, and we added this information in the revised manuscript.

**Comment:**

3. Fig. 4A and B lack statistical analysis.

**Response:**

The statistical analysis has been calculated and labeled in the revised manuscript.

**Comment:**

4. Page 10, line 3 "MycMycCorrespondingly," and line 4 "MycMyc-14-3-3η": Please correct.

**Response:**

We are sorry about this mistake. We corrected it in the revised manuscript.

**Comment:**

5. Page 6, "Sub-14-3-3η serves as ...." section, first paragraph: It would be informative to indicate the putative cleavage site to generate sub-14-3-3η is indicated using a tertiary structure of 14-3-3η.

**Response:**

Thanks to the reviewer’s suggestion. Indeed, this was what we considered when generating these constructs. We have included this info accordingly.

**Response to Reviewer #2:**

**Reviewer #2:** In this study, Chan et al. observed that 14-3-3n antibody stains a smaller band of the protein upon virus infection. In their prior work from 2019, they describe the role of 14-3-3n as a chaperone for MDA5. In this work, they find that after MDA5 activation, 14-3-3n is cleaved into a smaller molecular weight isoform that inhibits MDA5 function. They find that the cleavage depends on caspase-3 and residue D209. They then describe their finding that sub-14-3-3n exerts its activity in part by preventing MDA5 from localizing to the MAM, and this allows viruses that activate MDA5 to replicate better. From a host standpoint, sub-14-3-3u may be part of a negative feedback loop to prevent runaway inflammation.

Overall, the work and data presentation as figures and legends are clear. The manuscript has many typos and grammatical errors that sometimes interfere with the presentation of ideas.

**Major Issues:**

**Comment:**

Figure 2E it is very hard to tell that there is no sub-14-3-3η in the D209A lane due to the dark spots there. Possible to repeat, or maybe try probe/re-probing with 14-3-3 ab?

**Response:**

We have repeated the experiment and provided new Fig 2E with better presentation.

**Minor Issues:**

**Comment:**

Text throughout requires some copy editing to improve tenses and typos.

**Response:**

Thank the reviewer for the suggestion. We have made corrections in the revised manuscript.

**Comment:**

Introduction: grammar. Need to introduce 14-3-3 family and members better (just ε and η?

**Response:**

Thank the reviewer for the suggestion. We have corrected the grammar errors and increased the detailed background about 14-3-3 family.

**Comment:**

Figure 1C based on introduction, if cleavage of 14-3-3η is part of normal negative feedback, shouldn’t there be some cleaved 14-3-3η on a long exposure?

**Response:**

In figure 1C, the data did not show cleaved 14-3-3η. The cleaved 14-3-3η in poly IC stimulation cells may be degraded rapidly and therefore the sub-14-3-3η could not observed in this immunoblot.

**Comment:**

Figure 1D. On IB for Flag, why is MDA5 level different in TCL and in IP: Flag across conditions?

**Response:**

We understood the reviewer’s concern. The experiment was done by transfecting same Flag-MDA5 plamids amount in each samples co-transfected with different 14-3-3 isoforms to screen for MDA5-interacting 14-3-3 proteins. We are not comparing the MDA5 binding affinities among 14-3-3 proteins but to conclude that 14-3-3γ, 14-3-3η, and 14-3-3θ, but not 14-3-3β nor 14-3-3ɛ could interact with MDA5. Anti-Flag IP with equal amount of Flag-MDA5 was later done in Fig. 1E and 1F to compare the binding affinities.

**Comment:**

Figure 2C where is myc-MAVS? Mentioned in text but not figure or legend. Only in S2C? It would be helpful, wherever possible, to minimize chopping up the blots.

**Response:**

Myc-MAVS is in Fig S2C. Sorry for this mistake. We modified the text in the revised manuscript and tried to avoid chopping up the blots.

**Comment:**

Discussion: recommend a bit more robust discussion on other possible mechanisms of sub-14-3-3n inhibition of MDA5

**Response:**

Thank the reviewer for suggestion. We elaborated the possible molecular mechanism in more detail in discussion.
